# Applications of Robotic Surgery in Thoracic Diseases

**DOI:** 10.3390/jcm11144201

**Published:** 2022-07-20

**Authors:** Monica Casiraghi, Alessio Vincenzo Mariolo, Lorenzo Spaggiari

**Affiliations:** 1 Department of Thoracic Surgery, IEO, European Institute of Oncology IRCCS, 20141 Milan, Italy; lorenzo.spaggiari@ieo.it; 2 Department of Oncology and Hemato-Oncology, University of Milan, 20122 Milan, Italy; 3 Thoracic Surgery Department, Institut du Thorax Curie-Montsouris, Institut Mutualiste Montsouris, 75014 Paris, France; alessio.mariolo@imm.fr

With the ever-expanding implement of screening programs, as well as a raised awareness of patients about their own health, the number of cases of early-stage lung cancer is progressively increasing, leading surgeons to adapt their practice and to develop new surgical techniques that are less and less invasive. Thus, the scenario of thoracic surgery has been revolutionized in the last two decades by the development and assimilation of minimally invasive techniques such as video-assisted thoracic surgery (VATS) and robotic-assisted thoracic surgery (RATS). Despite the minimally invasive approach to thoracic surgery already having proven advantages in terms of reduced postoperative pain, shorter immune response, quicker resumption of daily activities, and better aesthetic and functional result [1,2,3,4,5,6], VATS lobectomy slowly became the standard approach to early-stage lung cancer treatment, probably due to its technical limitations, such as two-dimensional vision, lack of instrument flexibility with difficult hand–eye coordination, and a long-lasting learning curve, in particular for performing radical mediastinal lymphadenectomy, which is the standard treatment for lung cancer [7,8,9,10,11,12] is and highly related to the long-term outcome. To address these limitations, a tele-surgical system was developed offering surgeons the benefits of three-dimensional, high-definition imaging, greater hand movements using wristed instruments, and a computer-assisted scaling down of motion with the reduction in hand-related tremors (da Vinci system, Intuitive Surgical, Sunnyvale, CA, USA), offering surgeons an innovative approach to lung cancer resection and staging, with a more precise dissection and theoretically better oncological results.

RATS was only introduced in the operating room for pulmonary resection in 2002, with the first preliminary reports on pulmonary resection published by Melfi and Giulianotti in the early 2000s, showing the clinical feasibility of the technique with encouraging results [13,14]. Since then, many other publications confirmed over the years the feasibility and safety of the robotic technique, comparable with VATS [15,16,17,18], and different robotic approaches have been described ranging from the use of three or four robotic arms, utility incision or CO2 insufflation, and different port placement [19,20]. Despite RATS gaining popularity in the thoracic surgery community as one of the possible minimally invasive techniques used for both mediastinal tumors and pulmonary resection, the majority of papers published were focused on technical aspects, analyzing its safety and feasibility, while little data was available about survival and oncological outcomes. In fact, one of the major criticisms of minimally invasive surgery is the inadequate mediastinal lymph node dissection compared to open surgery, and concern over inferior oncologic outcomes has contributed to the slow adoption of minimally invasive surgery techniques. In the last decade, different studies have demonstrated that robotic pulmonary resection is oncologically safe, allowing for excellent lymph node removal [21,22,23,24,25,26] thanks to the 3D vision and the wide range of high-precision movements, even greater than the human wrist, crucial in performing lymphadenectomy. Toosi et al. [22] recently showed that RATS allowed for an adequate lymphadenectomy with the detection of occult lymph node metastatic disease and a significant upstaging (14.8%) with similar oncologic outcomes compared to open radical lymphadenectomy. In a multicenter study published by Cerfolio [23], the median number of lymph nodes resected was 13 (5 N2 stations and 1 N1), with only a 3% cumulative incidence of local recurrence (ipsilateral operated chest). Interesting oncological and long-term results were already published by Park in 2012 [21], showing a 5-year OS rate of 80% with a median follow-up of 27 months, increased up to 91% and 88% in stage IA and IB, respectively. In 2019, we reported our 10 years’ experience in RATS, showing a 5-year stage-specific survival rate of 91.5% for stage I NSLC with a median follow-up of 29 months [24] and no differences when compare to open surgery for cN0 patients [25]. These data have allowed thoracic surgeons to go further, widening the selection mesh of patient candidates for robotic surgery and also including more advanced tumors, in particular after induction therapy. Cerfolio showed an excellent 62% of 5-year stage-specific survival for stage IIIA and 51% in patients undergoing IT [23]. Even in our preliminary results on patients with locally advanced NSCLC after induction therapy [27], lymph nodes resection and positivity were not significantly different (*p* = 0.96 and *p* = 0.57, respectively) between RATS and open surgery, and no difference was found for PFS (*p* = 0.16) or OS (*p* = 0.41), demonstrating that the early outcomes and oncological results of N2-patients after robotic lobectomy were similar to open surgery.

In conclusion, considering the advantages of minimally invasive surgery, RATS should be a valid alternative not only for early-stage NSCLC but also for more advance tumors, with comparable favorable prognosis to open surgery, when performed in expert hands.

## References

[1] Demmy T.L., Curtis J.J. (1999). Minimally invasive lobectomy directed toward frail and high-risk patients: A case-control study. Ann. Thorac. Surg..

[2] Hoksch B., Ablassmaier B., Walter M., Muller J.M. (2003). Complication rate after thoracoscopic and conventional lobectomy. Zent. Fur Chir..

[3] Nakata M., Saeki H., Yokoyama N., Kurita A., Takiyama W., Takashima S. (2000). Pulmonary function after lobectomy video-assisted thoracic surgery versus thoracotomy. Ann. Thorac. Surg..

[4] Nomori H., Ohtsuka T., Horio H., Naruke T., Suemasu K. (2003). Difference in the impairment of vital capacity and 6-minute walking after a lobectomy performed by thoracoscopic surgery, an anterior limited thoracotomy, an antero-axillary thoracotomy, and a posterolateral thoracotomy. Surg. Today.

[5] Yim A.P., Wan S., Lee T.W., Arifi A.A. (2000). lobectomy reduces cytokine responses compared with conventional surgery. Ann. Thorac. Surg..

[6] Li W.W., Lee R.L., Lee T.W., Ng C.S., Sihoe A.D., Wan I.Y., Arifi A.A., Yim A.P. (2003). The impact of thoracic surgical access on early shoulder function video-assisted thoracic surgery versus posterolateral thoracotomy. Eur. J. Cardiothorac. Surg..

[7] McKenna R.J., Wolf R.K., Brenner M., Fischel R.J., Wurnig P. (1998). Is VATS lobectomy an adequate cancer operation?. Ann. Thorac. Surg..

[8] Leschber G., Holinka G., Linder A. (2003). Video-assisted mediastinoscopic lymphadenectomy (VAMLA)—A method for systematic mediastinal lymph node dissection. Eur. J. Cardiothorac. Surg..

[9] Yim A.P., Landreneau R.J., Izzat M.B., Fung A.L., Wan S. (1998). Is video-assisted thoracoscopic lobectomy a unified approach?. Ann. Thorac. Surg..

[10] Daniels L.J., Balderson S.S., Onaitis M.W., D’Amico T.A. (2002). Thoracoscopic lobectomy a safe and effective strategy for patients with stage I lung cancer. Ann. Thorac. Surg..

[11] McKenna R.J., Houck W., Fuller C.B. (2006). Video-assisted Thoracic Surgery Lobectomy: Experience with 1100 cases. Ann. Thorac. Surg..

[12] Whitson B.A., D'Cunha J., Andrade R.S., Kelly R.F., Groth S.S., Wu B., Miller J.S., Kratzke R.A., Maddaus M.A. (2008). Thoracoscopic Versus Thoracotomy Approaches to Lobectomy: Differential Impairment of Cellular Immunity. Ann. Thorac. Surg..

[13] Melfi F.M., Menconi G.F., Mariani A.M., Angeletti C.A. (2002). Early experience withrobotic technology for thoracoscopic surgery. Eur. J. Cardiothorac. Surg..

[14] Giulianotti P.C., Coratti A., Angelini M., Sbrana F., Cecconi S., Balestracci T., Caravaglios G. (2003). Robotics in general surgery: Personal experience in a large community hospital. Arch. Surg..

[15] Swanson S.J., Miller D.L., McKenna R.J., Howington J., Marshall M.B., Yoo A.C., Moore M., Gunnarsson C.L., Meyers B.F. (2014). Comparing robot-assisted thoracic surgical lobectomy with conventional video-assisted thoracic surgical lobectomy and wedge resection: Results from a multihospital database (Premier). J. Thorac. Cardiovasc. Surg..

[16] Mahieu J., Rinieri P., Bubenheim M., Calenda E., Melki J., Peillon C., Baste J.M. (2015). Robot-Assisted Thoracoscopic Surgery versus Video-Assisted Thoracoscopic Surgery for Lung Lobectomy: Can a Robotic Approach Improve Short-Term Outcomes and Operative Safety?. Thorac. Cardiovasc. Surg..

[17] Novellis P., Bottoni E., Voulaz E., Cariboni U., Testori A., Bertolaccini L., Giordano L., Dieci E., Granato L., Vanni E. (2018). Robotic surgery, video-assisted thoracic surgery, and open surgery for early-stage lung cancer: Comparison of costs and outcomes at a single institute. J. Thorac. Dis..

[18] Casiraghi M., Mariolo A.V., Mohamed S., Sedda G., Maisonneuve P., Mazzella A., Iacono G.L., Petrella F., Spaggiari L. (2022). Long-Term Outcomes of Robotic-Assisted, Video-Assisted and Open Surgery in Non-Small Cell Lung Cancer: A Matched Analysis. J. Clin. Med..

[19] Parini S., Massera F., Papalia E., Baietto G., Bora G., Rena O. (2022). Port Placement Strategies for Robotic Pulmonary Lobectomy: A Narrative Review. Clin. Med..

[20] Casiraghi M., Galetta D., Spaggiari L. (2018). Robotic assisted lobectomy and lymphadenectomy “different approaches”. Shanghai Chest.

[21] Park B.J., Melfi F., Mussi A., Maisonneuve P., Spaggiari L., Da Silva R.K., Veronesi G. (2012). Robotic lobectomy for non-small cell lung cancer (NSCLC): Long-term oncologic results. J. Thorac. Cardiovasc. Surg..

[22] Toosi K., Velez-Cubian F.O., Glover J., Ng E.P., Moodie C.C., Garrett J.R., Fontaine J.P., Toloza E.M. (2016). Upstaging and survival after robotic-assisted thoracoscopic lobectomy for non-small cell lung cancer. Surgery.

[23] Cerfolio R., Ghanim A.F., Dylewski M., Veronesi G., Spaggiari L., Park B.J. (2017). The long-term survival of robotic lobectomy for non–small cell lung cancer: A multi-institutional study. J. Thorac. Cardiovasc. Surg..

[24] Casiraghi M., Galetta D., Borri A., Tessitore A., Romano R., Diotti C., Brambilla D., Maisonneuve P., Spaggiari L. (2017). Ten Years’ Experience in Robotic-Assisted Thoracic Surgery for Early Stage Lung Cancer. Thorac. Cardiovasc. Surg..

[25] Spaggiari L., Sedda G., Maisonneuve P., Tessitore A., Casiraghi M., Petrella F., Galetta D. (2019). A Brief Report on Survival After Robotic Lobectomy for Early-Stage Lung Cancer. J. Thorac. Oncol..

[26] Gallina F.T., Tajè R., Forcella D., Corzani F., Cerasoli V., Visca P., Coccia C., Pierconti F., Sperduti I., Cecere F.L. (2022). Oncological Outcomes of Robotic Lobectomy and Radical Lymphadenectomy for Early-Stage Non-Small Cell Lung Cancer. J. Clin. Med..

[27] Casiraghi M., Petrella F., Sedda G., Mazzella A., Guarize J., Maisonneuve P., De Marinis F., Spaggiari L. (2021). Preliminary Results of Robotic Lobectomy in Stage IIIA-N2 NSCLC after Induction Treatment: A Case Control Study. J. Clin. Med..

